# Systematic identification of the lysine lactylation in the protozoan parasite *Toxoplasma gondii*

**DOI:** 10.1186/s13071-022-05315-6

**Published:** 2022-05-24

**Authors:** Wei Zhao, Helin Yu, Xiaona Liu, Tingting Wang, Yinning Yao, Qixin Zhou, Xiaozi Zheng, Feng Tan

**Affiliations:** 1 Department of Parasitology, School of Basic Medical Sciences, Wenzhou Medical University, Wenzhou, Zhejiang 325035 People’s Republic of China; 2 Teaching Center of Morphological Experiment, School of Basic Medical Sciences, Wenzhou Medical University, Wenzhou, Zhejiang 325035 People’s Republic of China

**Keywords:** *Toxoplasma gondii*, Lysine lactylation, Post-translational modification (PTM), Lactate, Tachyzoite

## Abstract

**Background:**

Lysine lactylation (Kla) is a novelposttranslational modification (PTM) identified in histone and nonhistone proteins of several eukaryotic cells that directly activates gene expression and DNA replication. However, very little is known about the scope and cellular distribution of Kla in apicomplexan parasites despite its significance in public and animal health care.

**Methods:**

*Toxoplasma gondii*, the causative agent of toxoplasmosis, is an obligate intracellular apicomplexan parasite that can infect different nucleated cell types of animals and humans. We used this parasite as a model organism and extracted the total protein of tachyzoites to produce the first global lysine lactylome profile of *T. gondii* through liquid chromatography–tandem mass spectrometry. We also investigated the level and localization of the Kla protein in *T. gondii* using western blotting and the indirect fluorescent antibody test (IFA), respectively.

**Results:**

A total of 983 Kla sites occurring on 523 lactylated proteins were identified in the total protein extracted from *Toxoplasma* tachyzoites, the acute toxoplasmosis-causing stage. Bioinformatics analysis revealed that the lactylated proteins were evolutionarily conserved and involved in a wide variety of cellular functions, such as energy metabolism, gene regulation and protein biosynthesis. Subcellular localization analysis and IFA results further revealed that most of the lactylated *T. gondii* proteins were localized in the nucleus, indicating the potential impact of Kla on gene regulation in the *T. gondii* model. Notably, an extensive batch of parasite-specific proteins unique to phylum Apicomplexa is lactylated in *T. gondii*.

**Conclusions:**

This study revealed that Kla is widespread in early dividing eukaryotic cells. Lactylated proteins, including a batch of unique parasite proteins, are involved in a remarkably diverse array of cellular functions. These valuable data will improve our understanding of the evolution of Kla and potentially provide the basis for developing novel therapeutic avenues.

**Graphical Abstract:**

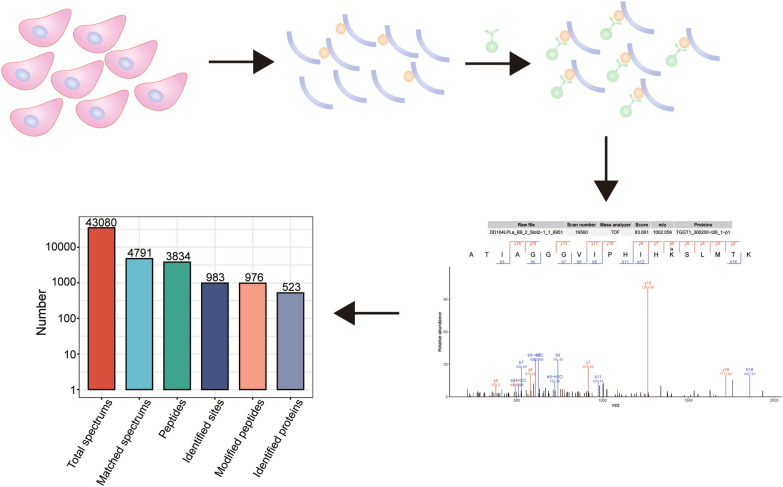

**Supplementary Information:**

The online version contains supplementary material available at 10.1186/s13071-022-05315-6.

## Background

The physicochemical properties, space conformation and stability of proteins after translation are regulated through protein posttranslational modification (PTM), a process of dynamic and reversible chemical modifications to the protein [1, 2]. The lysine residue is one of the most frequently modified targets due to its function in the organization of protein spatial structure. Lysine lactylation (Kla) is a novel PTM that has been identified on both histone and nonhistone proteins in several organisms, including humans [3, 4], mice [5], rice (*Oryza sativa*) [6], plant fungal pathogen (*Botrytis cinerea*) [7] and parasitic protozoan (*Trypanosoma brucei*) [8]. During this modification, the 72.021-Da of the l-lactate-derived lactyl group is covalently attached onto lysine residues via an enzymatic reaction mediated by the acetyl transferase p300 [3]. l-Lactate is an important and abundant metabolite produced through glycolysis [9, 10]. The level of Kla can be enhanced by increasing the intracellular concentrations of lactate in a dose-dependent manner. In contrast, inhibition of the glycolysis pathway and reduction of the level of exogenous sources (e.g. glucose) result in a decrease in the level of Kla [3, 5]. In humans and mice, Kla is enriched in the gene promoter regions lacking lysine acetylation (Kac) modification and is involved in regulating gene expression and DNA replication [3, 5].

Apicomplexan parasites including *Plasmodium* spp. and *Toxoplasma gondii*, the causative agents of malaria and toxoplasmosis, respectively, are important challenges in public and animal health care [11]. *Toxoplasma gondii* is an obligate intracellular protozoan parasite that infects most types of nucleated cells in animals and humans. Its hosts, which include humans and warm-blooded animals, can be infected by ingesting oocysts in contaminated food and water or tissue cysts in raw meat. Infection with this parasite can be lethal in immunocompromised individuals (e.g. those with AIDS, cancer patients and organ transplant recipients) [12]. The parasite can also infect the developing fetus of the host through transmission via the placenta during pregnancy; in particular, in pregnant women initially exposed during their first trimester, *T. gondii* infection may lead to several serious congenital disorders, such as miscarriage, fetal deformities and neurological pathologies [13]. Although most immunocompetent individuals are asymptomatic, *Toxoplasma* infection is associated with neurological disorders that can cause complications or even death in some cases [14, 15].

In addition to its biomedical significance, *T. gondii* has become an ideal model organism for other related parasites due to the advantages of genetical tractability, continuous culture in vitro and the ease of establishing a reliable mouse model of the disease. Within an intermediate host, *T. gondii* exists in both tachyzoite and bradyzoite stages, the causes of acute and chronic infection, respectively. As an obligate intracellular parasite, tachyzoites will secrete various proteins once they invade the host cells to establish a transient parasitophorous vacuole that enables them to avoid attack by host immune factors [16]. Consequently, tachyzoites need to salvage nutrients from the host cytosol to support their rapid replication throughout the acute stage of infection [17]. Tachyzoites can utilize both glucose and glutamine acquired from host cells for ATP production via glycolysis [18, 19]. These energy sources are metabolized to generate pyruvate, which is then either transported into the mitochondrion for ATP production by oxidative phosphorylation via the tricarboxylic acid cycle [20] or metabolized to form l-lactate [21]. It used to be thought that intracellular l-lactate must be exported in symport with H^+^ and that the impairment of the transport pathway would cause l-lactate to accumulate intracellularly, thereby threatening pH regulation and/or osmotic stability. *Toxoplasma gondii* harbors three formate nitrite transporters (TgFNTs) that are localized on parasite’s plasma membrane and responsible for transporting l-lactate [22, 23]. However, genetic disruption studies have provided evidence that each of the TgFNTs are dispensable during the lytic cycle [23]. In contrast to its early depiction as a metabolic waste product, as mentioned above, l-lactate is the principal messenger involved in lysine lactylation.

In this study, we first investigated the protein lactylation levels in intracellular and extracellular tachyzoites to evaluate the influence of host cell-derived lactate and to examine if the protein lactylation landscape changes as the tachyzoites transition from the intracellular to extracellular environment. We then used an integrated proteome-wide method to systematically identify the lysine lactylome of intracellular tachyzoites for further characterizing the mechanism of the metabolic regulation of gene expression by Kla in *T. gondii* tachyzoites.

## Methods

### Parasite culture and purification

Confluent human foreskin fibroblast cell (HFF) monolayers cultured in D10 medium (Dulbecco’s modified Eagle medium [DMEM] supplemented with 10% fetal bovine serum [FBS; Gibco™, Thermo Fisher Scientific, Waltham, MA, USA]) were infected with *T. gondii* RH Δku80Δhx tachyzoites and subsequently maintained in D1 medium (DMEM containing 1% FBS). When approximately 95% of host cells had been lysed, fresh extracellular tachyzoites were purified from the host cells’ debris by passage through 3.0-μm filters until no intact human cells were visible under the microscope. Intracellular tachyzoites were harvested at a density of approximately 64–128 parasites/vacuole, physically separated from host cells by passage through 27G needles and purified by passage through 3.0-μm filters. To harvest approximately 2 mg of tachyzoite total protein needed for mapping the lactylome, protein extracted from at least four independent biological replicates was pooled together.

### Protein extraction and lactylated peptide enrichment

The parasites were washed with cold phosphate-buffered saline, resuspended in lysis buffer (1% SDS, 1% protease inhibitors, 1% phosphatase inhibitor, 3 μM trichostatin A and 50 mM nicotinamide) and sonicated on ice for 3 min, with pauses for 3 s per 3 s of sonication for an intensity of 30%. Sonicated lysates were centrifuged at 16,000 *g* for 20 min at 4 °C*.* The collected protein supernatants were added successively to 1 volume and 4 volumes of pre-cooled acetone for precipitation at 4 °C for 2 h. The air-dried protein precipitate was resuspended in 200 mM TEAB and digested overnight with trypsin (Promega, Madison, WI, USA) at a 1:50 trypsin-to-protein mass ratio at 37 °C. The digested peptides were reduced with 5 mM dithiothreitol at 56 °C for 30 min and alkylated with 11 mM iodoacetamide at room temperature for 15 min in the dark. The alkylated peptide lysates were then resolubilized in IP buffer (50 mM Tris–Cl, 0.5% NP-40, 100 mM NaCl and 1 mM EDTA pH 8.0) and mixed with anti-l-lactyllysine antibody-conjugated agarose beads (PTM-1404; PTM Bio., Hangzhou, China) at 4 °C overnight with gentle shaking. The beads were washed 4 times with IP buffer and rinsed twice with water. Peptides were eluted from the beads using 0.1% trifluoroacetic acid and dried under vacuum. The dried peptides were desalted using C18 ZipTips (Merck Millipore, Burlington, MA, USA).

### Liquid chromatography–tandem mass spectrometry analysis

The desalted peptides were dissolved in the mobile phase A (0.1% formic acid and 2% acetonitrile) of the liquid chromatography (LC) chamber and separated using the NanoElute nano-flow UHPLC system (Bruker Corp., Billerica, MA, USA). The gradient consisted of a 7–24% increase in mobile phase B (0.1% formic acid and 100% acetonitrile) for 42 min, 24–32% for 12 min, 32–80% for 3 min and 80% for 3 min, at a constant flow rate of 450 nl/min. The separated peptides were injected into the capillary ion source for ionization and then into the timsTOF Pro mass spectrometer (Bruker Corp.) for analysis. The ion source voltage was set at 1.65 kV, and the peptide precursor ions and their secondary fragments were detected using high-resolution time-of-flight imaging. The scanning range of the secondary mass spectrum was set at 100–1700. The parallel accumulation serial fragmentation (PASEF) scan mode was used as the data acquisition mode. Notably, the second-level spectra with parent ion charges in the range of 0 and 5 were collected in the PASEF mode 10 times prior to collection of a first-level mass spectrum, with the dynamic exclusion time of the tandem mass spectrum scan set at 30 s to avoid repeated scans of the parent ion.

### Database search

The mass spectrometry (MS) proteomics data have been deposited to the ProteomeXchange Consortium via the PRIDE [24] partner repository with the dataset identifier PXD031526. The lactylated protein and sites were identified using MaxQuant software (v. 1.6.15.0). The MS/MS data were sequentially searched against the *T. gondii* protein subset of ToxoDB 54.0 (8460 sequences; release 2021.9.8; www.toxodb.org) and concatenated with a reverse decoy database and protein sequences of common contaminants. For the MaxQuant database searches, trypsin/P was adopted as the cleavage enzyme, with up to two missed cleavages allowed. The mass tolerance for precursor ions was set at 10 ppm in the first search and 5 ppm in the main search. The mass tolerance for fragment ions was set at 0.02 Da. Carbamidomethylation on cysteine was specified as a fixed modification, while lactylation modification and oxidation of methionine (Met) were specified as variable modifications. The false discovery rate (FDR) threshold was adjusted to < 1% and the minimum score for modified peptides was set at > 40.

### Bioinformatics analysis

Gene Ontology (GO) of lactylation proteome was derived from the ToxoDB 58.0 and UniProt-GOA database based on three categories: biological process, molecular function and cellular component. When a single peptide was found to be matched to ≥ 2 different proteins, manual inspection of data from ToxoDB 58.0 was performed to determine the protein from which the peptide was likely derived. The InterProScan software annotated the protein’s GO function of the identified Kla substrates not annotated by ToxoDB 58.0 and UniProt-GOA database based on protein sequence alignment. The InterPro domain database was used to annotate the identified proteins domain functional description with InterProScan based on protein sequence alignment. The Kyoto Encyclopedia of Genes and Genomes (KEGG) database description of identified proteins was annotated and mapped using KEGG online service tools KAAS and KEGG mapper. WoLF PSORT was used for subcellular localization prediction.

### Statistical analyses

#### Functional enrichment analysis

A two-tailed Fisher’s exact test was employed to test all functional enrichments of the differentially expressed proteins against all the identified proteins. Any terms with a corrected *P* < 0.05 in any of the clusters were treated as significant difference.

#### Sequence model analysis around the lactylation sites

The sequence models of lactylated proteins constituted with amino acids in specific positions of modify-21-mers (10 amino acids upstream and downstream of the lactylation site) in all protein sequences were surveyed using MoMo software (Motif-x algorithm). All protein sequences in the *T. gondii* database were used as background database parameters, with the other parameters set at default. The lowest possible number of events was set at 20, and the motif was detected at *P* < 0.000001.

#### Motif-based clustering analysis

All categories obtained after enrichment were collated along with their *P*-values, followed by filtering categories enriched in at least one of the clusters at *P* < 0.05. The filtered *P-*value matrix was transformed using the function *x* =  − log10 (*P-*value). The *x* values were then *Z*-transformed for each category to obtain *Z*-scores, which were then clustered by one-way hierarchical clustering (Euclidean distance, average linkage clustering) in Genesis. Cluster membership was visualized via a heat map constructed using the “heatmap.2” function of the “gplots” R-package.

#### Protein–protein interaction network

The protein–protein interaction (PPI) network was constructed using all the differentially expressed protein database accessions and sequences identified using the STRING database (v.10.1). Only PPIs belonging to the searched dataset with a confidence score of ≥ 0.7 were selected. The interaction network was visualized using the “networkD3” function in the R-package.

### Western blotting

Protein lysates extracted from the tachyzoites were electrophoresed on 10% (v/v) acrylamide gel and transferred onto a nitrocellulose membrane. Rabbit-derived monoclonal anti-l-lactyllysine antibodies (1:1000; PTM-1404RM; PTM BIO LLC, Chicago, IL, USA) were used as primary antibodies, while horseradish peroxidase-conjugated goat anti-rabbit IgG (1:2000) was used as the secondary antibody.

### Evaluation of the regulation of Kla by sodium lactate in *T. gondii* tachyzoites

HFFs infected with tachyzoites at a multiplicity of infection of 4:1 (parasite:host cell) were treated with sodium lactate (1, 5 and 25 mM) in D1 medium. Protein lysates from tachyzoites were then extracted 24 h post-treatment as described above for western blotting (WB).

### Evaluation of localization of lactated proteins in* T. gondii *tachyzoites

Immunofluorescence assays (IFA) were performed as described previously [25] to observe the localization of lactated proteins in tachyzoites. Primary antibodies used included the rabbit anti-l-lactyllysine (1:100) and mouse anti-TgSAG1 (1:500). The secondary antibodies used were the goat anti-rabbit Alexa Fluor 488 (1:1000; YEAST) and goat anti-mouse Alexa Fluor 594 (1:1000; YEAST). Images were acquired using the Eclipse Ci-L fluorescence microscope (Nikon, Tokyo, Japan) equipped with a CFI Plan Fluor optical imaging system, a 100 × /1.3-numerical-aperture (NA) Nikon lens, a digital sight camera connected to a PC via a DS-L4 control unit and NIS-Elements F 4.60.00 software. Brightness and contrast adjustments were applied uniformly to the entire image.

## Results

### Proteome-wide analysis of Kla in *T. gondii*

**Fig. 1:**
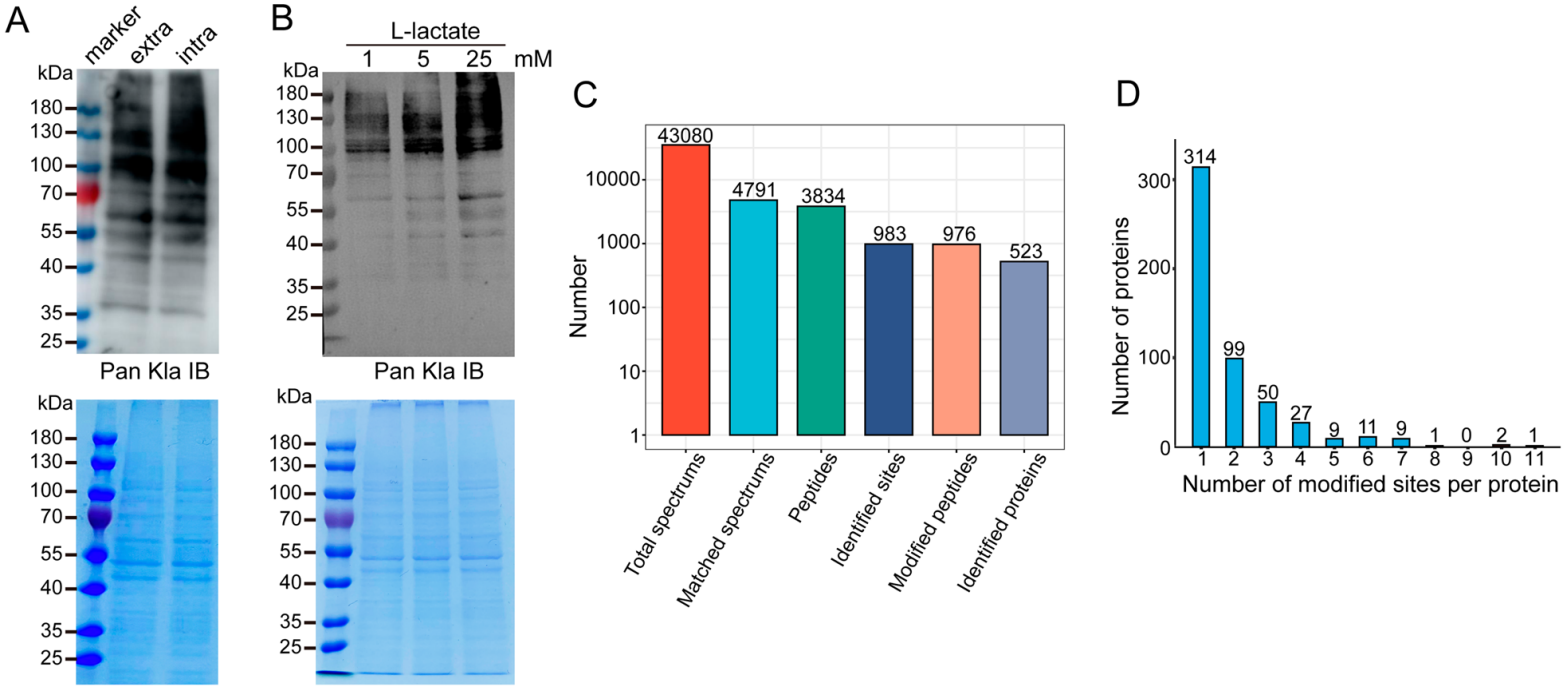
Overview of the *Toxoplasma gondii* lactylome. **A** WB analysis of tachyzoite lysate using lactyllysine antibody antibody demonstrate the presence of lactylated proteins in both extracellular (extra) and intracellular (intra) tachyzoites. The upper and lower panels show WB results and equal loading amounts by SDS-PAGE, respectively. **B** WB analysis of the regulatory effect of sodium lactate on intracellular parasites. The upper and lower panels show WB results and equal loading amounts by SDS-PAGE, respectively. **C** LC–MS/MS spectrum database search analysis summary. **D** Distribution of Kla proteins based on the number of modification sites. Abbreviations: IB, immunoblot; Kla, Lysine lactylation; LC-MS/MS, liquid chromatography-tandem mass spectrometry; SDS-PAGE, sodium dodecyl sulfate-polyacrylamide gel electrophoresis; Pan Kla,  pan-lactyllysine; WB, western blotting

To identify the presence of lactylated protein in *T. gondii*, extracellular and intracellular tachyzoites were collected, respectively, to compare the protein lactylation levels. As expected, WB with a rabbit monoclonal anti-l-lactyllysine antibody determined that various proteins with a wide range of molecular masses were lactylated, with Kla occurring relatively more frequently in intracellular tachyzoites (Fig. 1a). To identify the correlation between Kla level and lactate production, we subsequently treated the cultured parasites with l-lactate at final concentrations of 1, 5, and 25 mM prior to WB analysis. WB revealed that lactate treatment increased the Kla level in a dose-dependent manner (Fig. 1b).

To characterize the landscape of Kla in *T. gondii*, we performed global profiling of the lysine lactylome from intracellular tachyzoites. The distribution of mass errors was near zero, and the lengths of most lactylated peptides were in the range of 7–20 amino acids (Additional file 1: Figure S1), indicating that the tryptic peptide preparation and mass precision met the requirement for further analysis.

We obtained 43,080 total secondary ion spectra and 4791 matched spectra. A total of 3834 peptides and 976 lactylated peptides with a peptide score > 40 were identified in *Toxoplasma* tachyzoites. Additionally, 983 Kla sites across 523 proteins, accounting for about 6.3% (523/8460) of proteins, were identified with < 1% FDR by MaxQuant (Fig. 1c; Additional file 2: Table S1). Among the 523 lactylated proteins identified, approximately 60% (314/523) possessed a single lactylated site, 19% (99/523) possessed two lactylated sites and 10% (50/523) carried three identified sites. The average degree of lactylation was 1.9 (983/523). Notably, 33 proteins possessed ≥ 5 lactylated sites. The most extensively lactylated protein was the putative elongation factor 1-alpha (TGGT1_286420), which contains up to 11 independent lactylated lysine residues (Fig. 1d).

### Characterization of lysine lactylome in *T. gondii*

**Fig. 2:**
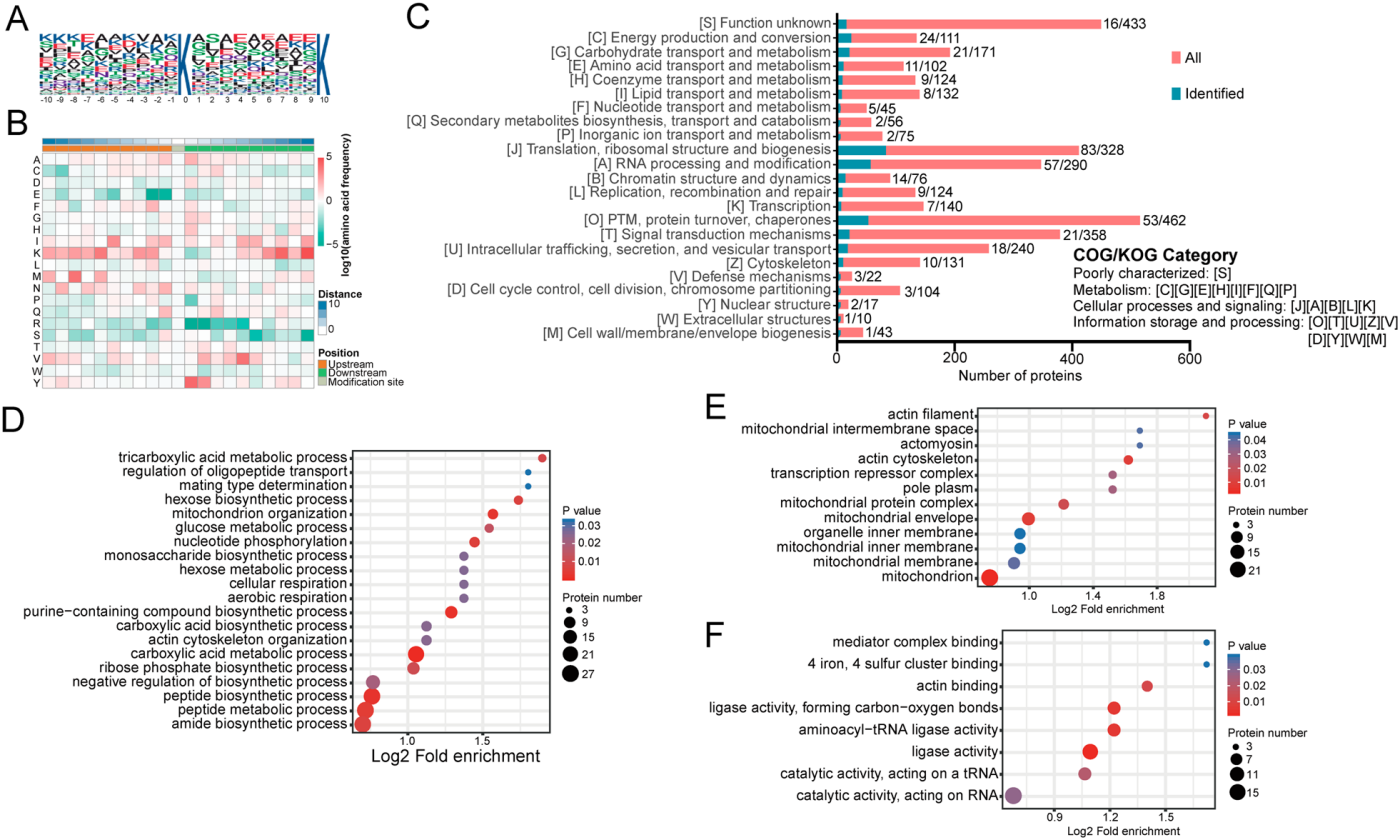
Characterization of lysine lactylome of *T. gondii*. **A** Probability sequence motifs of *Toxoplasma* lactylation sites consisting of 20 residues surrounding the targeted lysine residue. The central K (at position 0) indicates the lactylation lysine. All of the flanking amino acid residues are indicated with the letters differing in height, with the differences corresponding to their frequencies in the respective position. **B** Heat map showing the frequency of certain amino acids in specific positions flanking the lactylated lysine in *Toxoplasma*. Red and green indicates high frequency and low frequency, respectively.** C**–**F** KOG annotation functional classification of the identified lactylated proteins (**C**) and GO-based enrichment analysis of lactylated proteins according to biological process (**D**), cellular component (**E**) and molecular function (**F**). *P* < 0.05 indicates a significant difference. Abbreviations: COG, Cluster of Orthologous Groups of proteins database; KOG, Eukaryotic Orthologous Groups

To characterize the nature of Kla in *T. gondii*, we used the Motif-X program to conduct the motif analysis of amino acids flanking the Kla sites from the − 10 to + 10 positions (lactyl-21-mers). Motif analysis results revealed that of all the Kla peptides, 103 peptides included the amino acid sequence from the − 10 to the + 10 positions surrounding the Kla site (Fig. 2a; Additional file 3: Table S2). A survey of the motif revealed that lactylation had a strong substrate sequence preference for the negatively charged lysine (K) 10 amino acids downstream of the Kla sites (KlaxxxxxxxxxK, where x indicates a random amino acid residue;* P* < 0.000001). In the distinct motif, moreover, the frequency of E at the -2 or − 1 position, R at the + 1 or + 2 position and S at the + 10 position is the lowest, as determined by an inspection of the heat map of the amino acid composition surrounding the Kla site (Fig. 2B).

To further understand the biological regulations and functions of lactylated proteins in *T. gondii*, we classified these proteins based on Eukaryotic Orthologous Groups (KOG) annotation. According to this classification, most lactylated proteins were involved in translation, ribosomal structure and biogenesis, RNA processing and modification, as well as PTM, protein turnover and chaperones (Fig. 2c; Additional file 4: Table S3). Also, a significant number of proteins involved in energy production and conversion, carbohydrate transport and metabolism pathway were subjected to lactylation (Fig. 2c; Additional file 4: Table S3). GO annotation revealed that Kla occurred on diverse proteins involved in biological processes, cellular components and molecular function. We also performed functional enrichment analysis of GO. The biological process enrichment analysis showed that the majority of lactylated proteins were involved in mitochondrion organization (Fig. 2d; Additional file 4: Table S3). Based on the cellular component enrichment analysis, lactylated proteins were mostly located in the mitochondrion and actin cytoskeleton (Fig. 2e). These findings were supported by molecular function enrichment analysis in that many lactylated proteins were related to actin-binding and ligase activity (Fig. 2f).

**Fig. 3:**
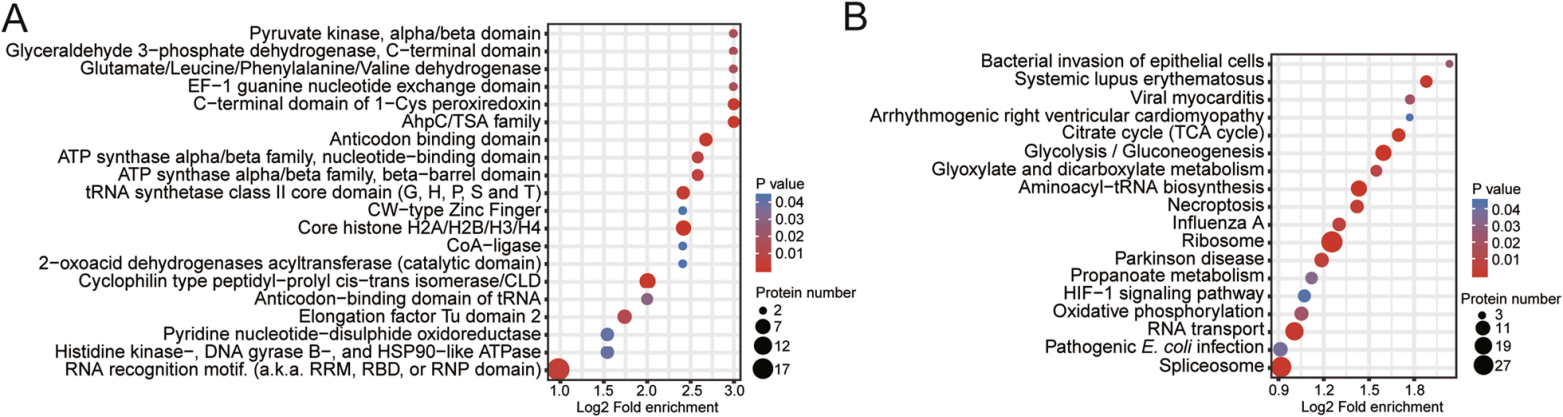
Domain and KEGG pathway enrichment analysis of Kla proteins in *T. gondii*. **A** Domain enrichment analysis of lactylated proteins. **B** Enriched KEGG pathway analysis for lactylated proteins. *P* < 0.05 indicates a significant difference. Abbreviations: KEGG, Kyoto Encyclopedia of Genes and Genomes

The domain enrichment analysis further revealed that RNA recognition motif, core histones and cyclophilin type peptidyl-prolyl* cis–trans* isomerase/CLD were highly enriched in the *T. gondii* lactylome (Fig. 3a). KEGG pathway enrichment analyses revealed that the lactylated proteins were associated with glycolysis, aminoacyl-transfer RNA (tRNA) biosynthesis and ribosome formation (Fig. 3b; Additional file 5: Table S4).

### Lactylation targets the metabolism pathway in *T. gondii*

**Fig. 4:**
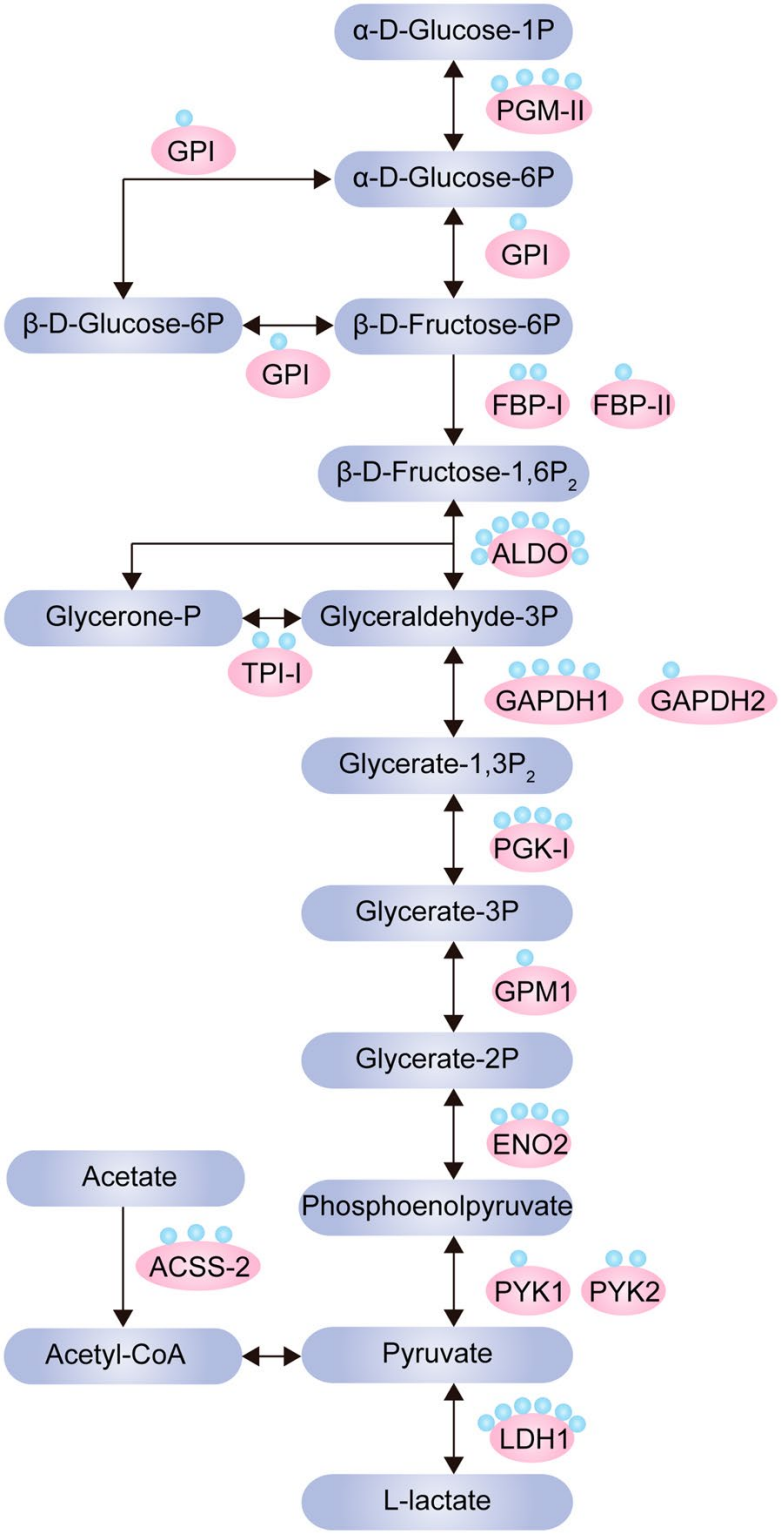
Lactylated enzymes involved in glycolysis in *T. gondii* tachyzoites. Green dots indicate proteins subjected to lactylation. K (red) indicates Kla sites. Abbreviations: PGM-II, Phosphoglycerate mutase-II (PGM-II); GPI, glucose-6-phosphate isomerase; FBP-I, fructose-bisphosphatase-I; FBP-II, fructose-bisphosphatase-II; ALDO, fructose-1,6-bisphosphate 1lase; TPI-I, triosephosphate isomerase-I; GAPDH1, glyceraldehyde-3-phosphate dehydrogenase 1; GAPDH2, glyceraldehyde-3-phosphate dehydrogenase 2; PGK-I, phosphoglycerate kinase-I; GPM1, glucosephosphate-mutase 1; ENO2, enolase 2; PYK1, pyruvate kinase 1; PYK2, pyruvate kinase 2; LDH1, lactate dehydrogenase 1; ACSS2, putative acetyl-coenzyme A synthetase 2

Glycolysis is the main energy metabolic pathway by which tachyzoites of *T. gondii* use both glucose and glutamine as energy sources for ATP production [18, 19]. KEGG enrichment analyses focused on the glycolysis pathway and revealed that most key enzymes involved in glycolysis were lactylated. A total of 44 Kla sites were identified on 15 glycolytic enzymes. Among these, aldolase (ALDO), lactate dehydrogenase1 (LDH1), enolase2 (ENO2, 4 sites), glyceraldehyde-3-phosphate dehydrogenase 1 (GAPDH1), phosphoglycerate mutase-II (PGM-II) and phosphoglycerate kinase-I (PGK-I) harbored at least four Kla sites (Fig. 4; Table 1).

**Table 1:** Identified Kla proteins involved in glycolysis pathway

Protein namea	ToxoDB accession no.	Product description	Kla site(s) (amino acids)
PGM-II	TGGT1_297060	Phosphoglycerate mutase-II	195, 189, 326, 337
GPI	TGGT1_283780	Glucose-6-phosphate isomerase	650
FBP-I	TGGT1_205380	Fructose-bisphospatase-I	15, 92
FBP-II	TGGT1_247510	Fructose-bisphospatase-II	70
ALDO	TGGT1_236040	Fructose-1,6-bisphosphate aldolase	177, 180, 393, 382, 385, 111, 229, 429
TPI-I	TGGT1_225930	Triosephosphate isomerase-I	196, 58
GAPDH1	TGGT1_289690	Glyceraldehyde-3-phosphate dehydrogenase 1	220, 405, 366, 211
GAPDH2	TGGT1_269190	Glyceraldehyde-3-phosphate dehydrogenase 2	897
PGK-I	TGGT1_318230	Phosphoglycerate kinase-I	139, 141, 92, 323
GPM1	TGGT1_285980A	Glucosephosphate-mutase1	285
ENO2	TGGT1_268850	Enolase 2	103, 112, 118, 437
PYK1	TGGT1_256760	Pyruvate kinase1	4
PYK2	TGGT1_299070	Pyruvate kinase2	328, 400
LDH1	TGGT1_232350	Lactate dehydrogenase 1	218, 120, 211, 312, 322, 307
ACSS2	TGGT1_266640	Putative Acetyl-coenzyme A synthetase 2	283, 328, 406

Kla, Lysine lactylation

### Lactylation targets histone proteins in *T. gondii*

**Fig. 5:**
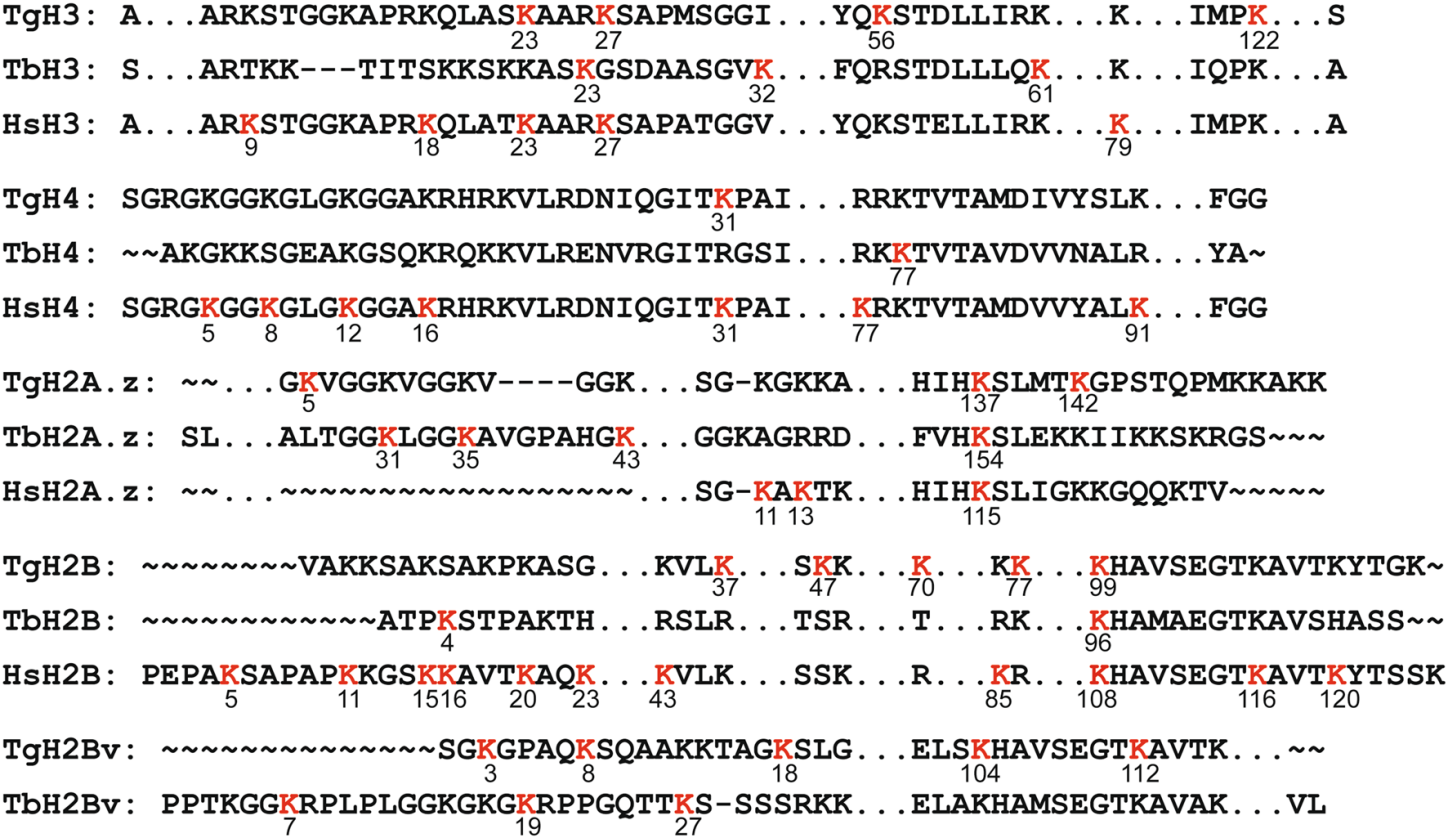
Comparison of lactylated residues in histone proteins of *T. gondii* (Tg) with that of *T. brucei* (Tb), and *H. sapiens* (Hs). Red sequences indicate the lactylation sites. Numbers below the sequences represent the amino acid position

Histone sites modified through lactylation have previously been identified in several eukaryotic species [3, 5–8] and were found to directly activate gene expression [3]. In the present study, a total of 20 Kla sites on *T. gondii* canonical and variant histones were identified, including five sites on H2B (K37, K47, K70, K77 and K99), four sites on H3 (K23, K27, K56 and K122), three sites on H2A (K5, K137 and K142) and one site on H4 (K31), H2A1 (K73) and H2AX (K127) (Fig. 5). Sequence alignment analysis suggests that the Kla site on either H2A.zK137 or H2BK99 in *T. gondii* was also present in humans [3] and *T. brucei* [8]. Lactylation on H3K23, H3K27 and H4K31 in *T. gondii* is also conserved in human cells at the same site [3]. Interestingly, no conserved Kla sites on H2Bv were observed between *T. gondii* and *T. brucei*, indicating that Kla sites on H2Bv are probably a species-specific modification.

### Lysine lactylation on Apicomplexa unique proteins

The *T. gondii* genome encodes a great diversity of proteins or protein families that lack clear homologs in metazoan species, such as proteins harboring a plant-like Apetela-2 (AP2) DNA binding domain [26] and secretory proteins produced from the rhoptry, microsome and dense granules [27]. In this study, we found that there were a few Kla modifications on proteins unique to apicomplexans (Table 2). The AP2 domain proteins were the first group of such proteins to be noted. Nine AP2 domain proteins in *T. gondii*, including AP2IX-8, AP2X-1, AP2X-4, AP2X-7, AP2XI-2, AP2XI-3, AP2XI-5 and AP2XII-1 were modified with Kla. Both AP2X-1 and AP2XI-2 harbored the most Kla sites (6 independent sites). In addition, we observed Kla on 10 secretory proteins, including ROP5, ROP9, ROP18, MIC1-4, GRA2, GRA3 and GRA12. Notably, there are 29 hypothetical parasite-specific proteins that were modified by Kla (Table 3).

**Table 2:** Kla proteins identified to be unique to phylum Apicomplexa

Protein name	ToxoDB accession no	Kla site(s) (amino acids)
*Proteins harboring AP2 DNA binding domain*		
AP2IX-8	TGGT1_306000	517
AP2X-1	TGGT1_227900	540, 1229, 1179, 455, 1169, 592
AP2X-4	TGGT1_224050	379
AP2X-7	TGGT1_214840	639, 1266
AP2XI-2	TGGT1_310900	1321, 1288, 1064, 1990, 2223, 78
AP2XI-3	TGGT1_310950	288, 1060, 1015
AP2XI-5	TGGT1_216220	187, 835
AP2XII-1	TGGT1_218960	142
AP2XII-5	TGGT1_247730	402, 323, 329
*Rhoptry proteins*		
ROP5	TGGT1_411430	118
ROP9	TGGT1_243730	144
ROP18	TGGT1_205250	202
*Microneme proteins*		
MIC1	TGGT1_291890	157
MIC2	TGGT1_201780	162, 624
MIC3	TGGT1_319560	156, 293, 83, 356
MIC4	TGGT1_208030	211, 74
*Dense granule proteins*		
GRA2	TGGT1_227620	52, 99, 88, 116
GRA3	TGGT1_227280	114
GRA12	TGGT1_288650	421, 74, 72, 357

**Table 3:** Kla identified on hypothetical parasite-specific proteins

ToxoDB accession no	Kla site(s) (amino acids)
TGGT1_202890	195
TGGT1_204490	56
TGGT1_209500	1352, 1763, 2434
TGGT1_211030	107, 148
TGGT1_211250	82
TGGT1_215350	75
TGGT1_219690	77
TGGT1_221470	374, 386
TGGT1_223610	680
TGGT1_226350	509
TGGT1_234230	1983, 1969, 1856, 4219, 4229
TGGT1_235340	151
TGGT1_236560	643
TGGT1_236820	278
TGGT1_244690	238
TGGT1_248800	680
TGGT1_250820	748
TGGT1_254800	36
TGGT1_261490	756
TGGT1_277710	578
TGGT1_289970	203
TGGT1_295370	136
TGGT1_299670	248, 67
TGGT1_305610	338
TGGT1_311810	225
TGGT1_314070	69
TGGT1_315570	6
TGGT1_315610	110
TGGT1_320015	150

### Localization and PPI network of Kla in *T. gondii*

**Fig. 6:**
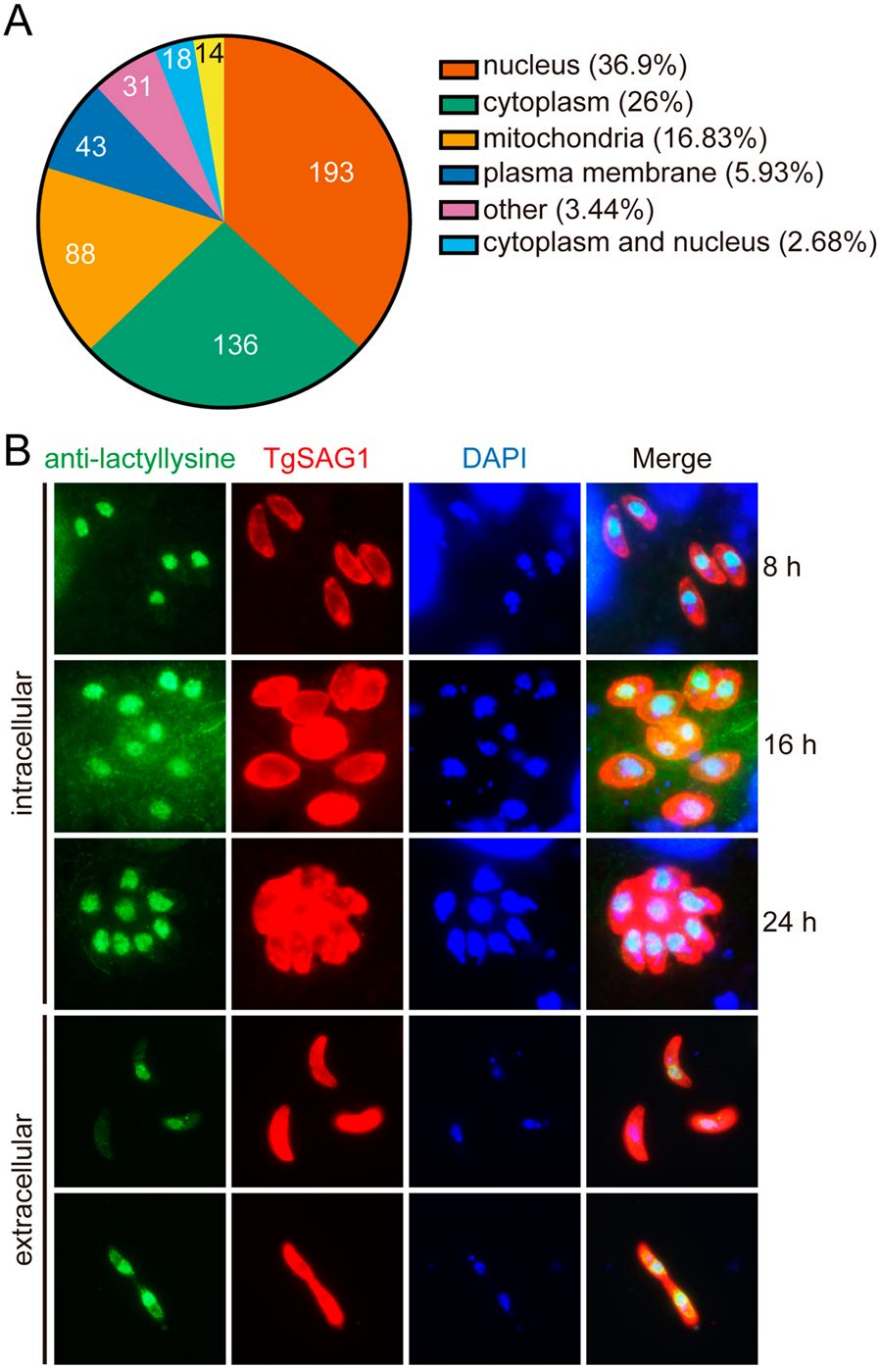
Subcellular localization of lactylated proteins in *T. gondii*. **A** Pie chart indicating subcellular lactylated protein localization. **B** Immunofluorescence assays of the lactylated protein localization in both intracellular and extracellular tachyzoites

To illustrate the subcellular distribution of the lactylated proteins in *T. gondii*, we annotated all of the lactylated proteins using predicted functions and localizations. The classification results showed that the lactylated *T. gondii* proteins were mainly distributed in the nucleus, with 36.9% (193/523) in the nucleoprotein and 2.68% (14/523) in both the cytoplasm and nucleus. In addition, 26% (136/523) of the lactylated proteins were cytoplasmic proteins, and 16.83% (88/523) were in the mitochondria (Fig. 6a; Additional file 6: Table S5). Subsequently, fluorescence microscopic imaging was used to detect the Kla signals in both intracellular and extracellular tachyzoites expressing the stage-specific marker TgSAG1 (Fig. 6b). The imaging results were consistent with the classification results that Kla modifications primarily occur in the nucleus.

**Fig. 7:**
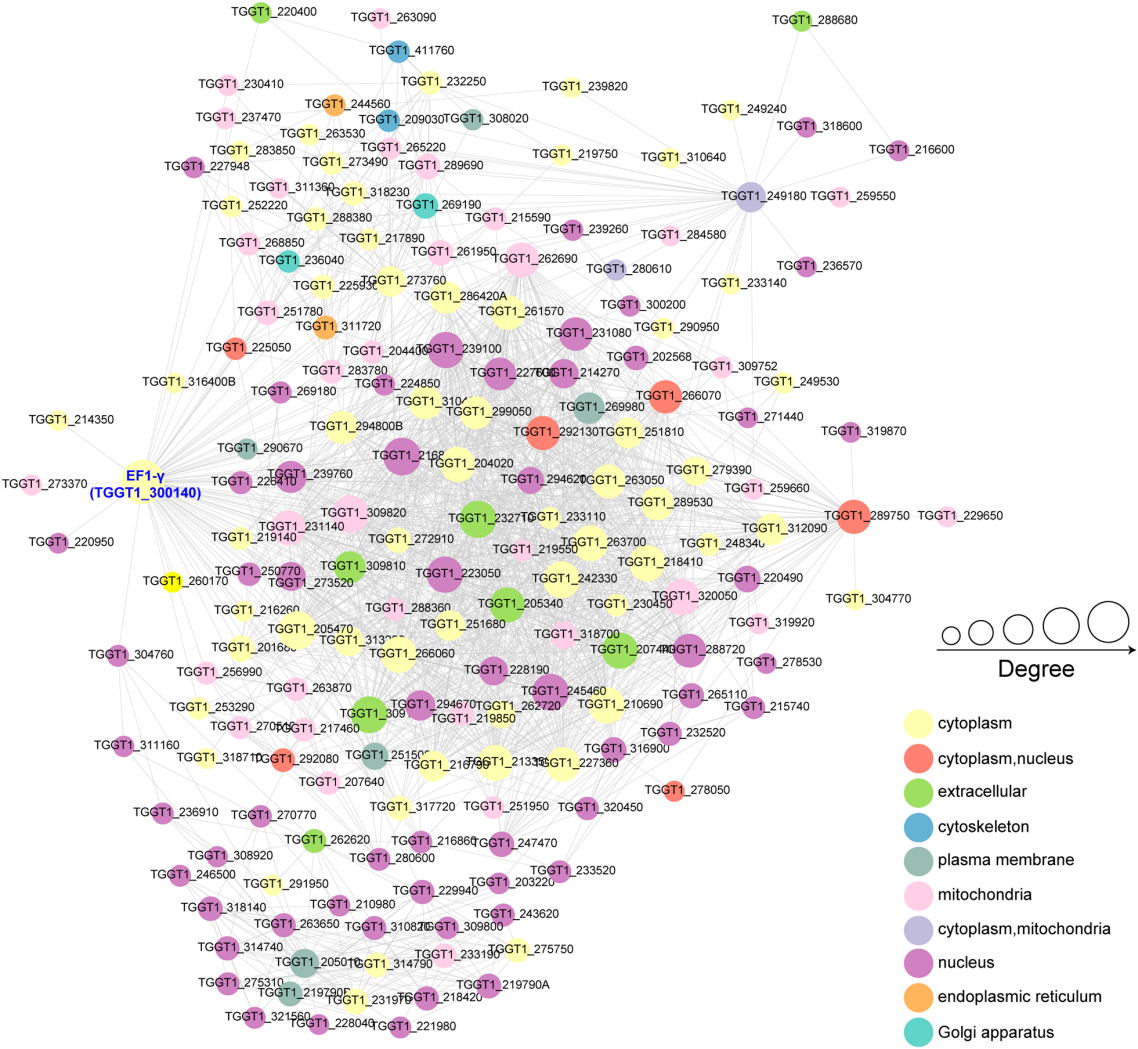
Protein–protein interaction networks for all Kla proteins in *T. gondii*. Interactive proteins are linked by lines. Different colors represent functional protein clusters based on KOG classification. Circle sizes are proportional to node degree

To better comprehend the cellular processes regulated by Kla *in T. gondii*, we further mapped the identified lactylated proteins onto the PPIs predicted in the STRING database. The PPI network, including a multitude of lactylated proteins, showed that translation and ribosomal structure proteins were the most prominently enriched cluster. This finding suggests that Kla modification is associated with altered control of the protein translational process, which is central to the generation of proteins. Among these proteins, a putative elongation factor 1-gamma (EF1-γ, TGGT1_300140) showed the strongest interactions with proteins related to translation and ribosomal structure (Fig. 7; Additional file 7: Table S6).

## Discussion

Lysine lactylation, a novel acylation type among various PTM processes, has been demonstrated through proteome-wide studies in several eukaryotic cells. In this study, we combined high-affinity enrichment of lysine lactylated peptides with high-sensitive MS and bioinformatics tools and have provided the first in-depth insight into the lysine lactylome for *T. gondii*, an apicomplexan parasite. We identified 983 lysine lactylation sites on 523 lactylated proteins in *Toxoplasma* tachyzoites. To identify the presence of lactylated protein in *T. gondii*, tachyzoite lysates were prepared for immunoblotting using an antibody recognizing lactyl-lysine. As expected, the analysis of the data revealed that a number of proteins with a wide range of molecular masses were lactylated.

In *T. gondii*, two tachyzoite phases, replicating intracellular tachyzoites and egressed extracellular tachyzoites, with differences in energy metabolism have been identified. Our comparison of intracellular and extracellular tachyzoites suggested that the intensities of the bands corresponding to intracellular tachyzoites were relatively stronger. However, this result is insufficient to prove that the level of protein lactylation in intracellular tachyzoites was higher than that in extracellular tachyzoites. Therefore, to further illuminate the biologic function of lactylation in *T. gondii*, and to explore whether the modification landscape changes as the tachyzoites transition from one environment to another, a further global lactylome analysis on non-replicating extracellular parasites is necessary. Nevertheless, multiple protein bands spanning a wide mass range were observed, suggesting the presence of non-histone Kla. Furthermore, to assess whether the lactylation level is regulated by the lactyl-CoA, the substrate of the Kla reaction, we treated intracellular tachyzoites with different concentrations of sodium lactate, which could be converted to lactyl-CoA. We noted that this exogenous lactate treatment increased total protein Kla levels in a dose-dependent manner, which is in consistent with previous findings in humans [3] and the mouse [5]. Given that lactylation occurs through the transfer of the l-lactate-derived lactyl group to substrate proteins, we predict that the extensive lactylation works as an alternative metabolism pathway for the regulation of intracellular lactate level; however, the roles of lactylation require further study.

A motif logo reflecting the relative frequency of amino acids in specific positions of lactyl-21-mers (10 amino acids upstream and downstream of the modification site) showed that a lysine residue at the +10 position in the *T. gondii* lactylome was the preferred lysine lactyltransferase substrate. The bias for the amino acid may represent a genuine preference or be attributed to the sequence bias of anti-lactyl lysine antibodies used for selective enrichment of lactylated peptides, as previously described for acetyl-lysine enrichment in *T. gondii* [28]. Although p300 has been identified as a regulatory enzyme for mediating lysine lactylation [3, 29], acetylation [30] and succinylation [31] in mammals, its homolog encoded in the *T. gondii* genome has not been identified yet, suggesting the presence of different enzymes engaged in establishing the modification. Interestingly, Kla was identified on two histone deacetylases, HDAC3 (K433) and HDAC5 (K34). It would therefore be of value to further investigate whether they are involved in the removal of Kla modification in *T. gondii*.

Cellular metabolism is associated with the generation of energy and the synthesis of complex biomolecules through nutrient uptake, release and interconversion. It has been determined that many metabolic products, including intermediates and end products, regulate cell signaling and gene expression depending on nutrient availability. The tachyzoite is a rapid replication and disease-causing stage of *T. gondii* that primarily utilizes glucose for ATP production via glycolysis metabolism to maintain parasite survival and virulence. Traditionally, studies found that l-lactate is the major waste product of glycolysis and must be exported from cells. In *Plasmodium falciparum*, the formate nitrite transporter (PfFNT) that is localized to the parasite’s plasma membrane and involved in transporting l-lactate across the plasma membrane has been validated as an antimalarial drug target. It is now evident that *T. gondii* tachyzoites express all of the enzymes needed for complete glycolysis and also harbor three plasma membrane-localized FNTs that are responsible for transporting l-lactate [22, 23]. However, all three *TgFNT* genes can be genetically disrupted alone and together without impairing the lytic cycle of the tachyzoite [23]. Combining these findings and our lactylome results, we predict that the surplus l-lactate in tachyzoites may act as a regulator in glycolysis and other metabolic pathways, thereby enabling the parasite to avoid the cytotoxicity caused by lactate accumulation. It is thus worth investigating if lactylation-regulated glycolytic enzymes have any effect on the glycolysis pathway and energy production, particularly in terms of the role of the lactate shuttling in metabolic signaling, by eliminating the Kla modification through lysine point mutation.

We found more lactylated proteins and sites in *T. gondii* than have been reported for the parasite *Trypanosoma brucei*, in which 387 Kla sites occurring on 257 lactylated proteins were identified [8]. The distinction in lactate dehydrogenase (LDH), which catalyzes the synthesis of lactate, possibly contributes to differences in Kla levels between the two parasites, given that the *T. gondii* genome encodes two LDH enzymes (TgLDH1 and TgLDH2) [32, 33], while trypanosomes lack LDH [34, 35]. One unique characteristic of *T. gondii* is its ability to interconvert between acute and chronic stages, known as the tachyzoite and bradyzoite, respectively, which is significantly associated with the pathogenesis and transmission of this parasites [36]. Interesting, in *T. gondii*, most of the glycolytic enzymes have two isoforms which are differentially expressed in both the tachyzoite and bradyzoite [37]. Among these, LDH and enolase (ENO) are the most widely studied. TgLDH1 and TgENO2 are principally expressed in tachyzoites, while TgLDH2 and TgENO1 are almost exclusively produced in bradyzoites. Additionally, both TgENO1 and TgENO2 can act as nuclear factors to regulate the expression of a number of genes by shuttling between the nucleus and cytoplasm. In addition, previous studies revealed that pyruvate kinase and TgLDH activities were significantly higher in bradyzoites [38] and that inhibition of mitochondrial function could promote expression of bradyzoite-specific genes [39]. These results suggest that bradyzoites rely mainly on anaerobic glycolysis for energy production. Since our functional annotation analysis highlights that lactate plays an importance role in linking glycolysis metabolism to gene expression in *T. gondii*, it would thus be valuable to further investigate the regulatory functions of lactylation of these enzymes in the interconversion between the tachyzoite and bradyzoite.

IFA assays and subcellular localization analysis revealed that lactylated proteins in *T. gondii* were relatively more abundant in the nucleus. Due to its complex life-cycle, *Toxoplasma* needs to regulate gene expression in response to a variety of stimuli with a precise and fast regulatory system, such as epigenetic gene regulation. The key role of histone modification in epigenetic gene regulation has been validated [40]. Understanding the function of histone lactylation in cellular physiology may thus increase the potential for developing new antiparasitic drugs. Core *T. gondii* histones consist of a single copy of H2A, H3 and H4, as well as two isoforms of H2B (H2Ba and H2Bb) [40]. H2A and H2B are more divergent, while H3 and H4 are highly conserved. Through MS analysis, we identified 20 Kla sites on *T. gondii* histones, which are fewer than those reported in humans (26 histone Kla sites) but more than those in mice (16 histone Kla sites) and *T. brucei* (16 histone Kla sites). Notably, while *T. gondii* H4 differs from human H4 by only six amino acids, only one residue (TgH4K31) was identified for Kla modification on *T. gondii* H4, which is much fewer than the six Kla sites on human H4. These differences highlight the distinct function of Kla modification on histones among different species or a limitation linked with the affinity of the anti-lactyl antibody used for peptide enrichment in this study. Intriguingly, H3K122 identified in this study is a novel histone lactylation that has not been described in other species. The lactylation of H3 at K122 may thus be specific to *T. gondii* and may serve as a marker for species identification. However, histone lactylation is able to change dynamically with different external stimuli and environmental changes. Given that *T. gondii* must pass through different hosts to survive, it is likely that the parasite responds to environmental changes and external stimuli cues by regulating histone lactylation, which in turn alters the chromatin structure and gene expression. Quantitative measurement of histone lactylation changes during developmental transitions is thus a subject for future research.

Instead of the canonical transcription factors conserved in most eukaryotes, all apicomplexans seem to regulate targeted gene expression through a branch of proteins containing an AP2 DNA-binding domain. In this study, we identified 25 Kla sites across nine AP2 proteins in *T. gondii.* Of note, all Kla sites were located outside the DNA-binding domain except four (AP2IX-8K517la, AP2XI-3K1060la, AP2XII-5K323 and AP2XII-5K329). Lysine acetylation adjacent to the DNA-binding domain enhances DNA binding, while lysine acetylation within the domain hinders DNA binding [41, 42]. However, it is unclear if Kla directly impacts the interaction between DNA and protein. A battery of important virulence factors is secreted by parasites into host cells during invasion to regulate the host immune response. Although we observed 21 Kla sites on 10 secreted proteins, where these modifications occur and their corresponding functions should also be further studied. Taking into considereation the many identified hypothetical parasite-specific proteins, the findings of this study indicate that the evolution of Kla modification follows species-specific trajectories and further suggest that the Kla proteins may hold a great promise for new therapeutic opportunities. Finally, we found that the putative eukaryotic translation TgEF1-γ strongly interacted with other proteins related to translation and ribosomal structure. Based on our integration of the results of KOG annotation analyses showing that most lactylated proteins were involved in translation, ribosomal structure and biogenesis, we are of the opinion that lactylation in *T. gondii* plays an important and special role in translation and ribosomal biogenesis. However, the detailed mechanism by which the lactylation on TgEF1-γ regulates protein translation and ribosomal biogenesis remains to be further verified.

## Conclusions

This study is the first to systematically delineate the lactyl-proteome profile of *T. gondii*. Kla modification of *Toxoplasma* proteins involved in diverse metabolic processes and biological regulation occurs on a large scale, highlighting the significance of protein lactylation in regulating *T. gondii* physiology. These findings enabled us to demonstrate the function and abundance of lactylation in *T. gondii*, thereby increasing our understanding of the relationship between glucose metabolism and gene regulation in *T. gondii* and other members of the Apicomplexa phylum. They also form a basis for discovering potential drug targets.

## Supplementary Information

**Additional file 1: Fig. S1.** Quality control of lactylated proteins in *T. gondii*. Scatter plot and bar plot show the peptide mass tolerance distribution and the length distribution of peptides identified by mass spectrometry, respectively.

**Additional file 2: Table S1.** Identified lactylated proteins and the Kla sites in *T. gondii.*

**Additional file 3: Table S2.** List of peptides matches the enriched motif in *T. gondii.*

**Additional file 4: Table S3.** Function classification by Clusters of Orthologous Groups (COG) of lactylated proteins in *T. gondii.*

**Additional file 5: Table S4.** KEGG pathway enrichment in *T. gondii.*

**Additional file 6: Table S5.** Subcellular distribution of the lactylated proteins in T. gondii. 

**Additional file 7: Table S6. **PPI network of Kla in T. gondii.

## Data Availability

The raw data and annotated MS spectra of this article are available in the proteomics repository PRIDE with a ProteomeXchange (http://proteomecentral.proteomexchange.org) dataset accession number of PXD. For reviewing, the username is reviewer_pxd123@ebi.ac.uk and the password is 6VwjhF3z.

## Abbreviations

AP2: Apetela-2
DMEM: Dulbecco’s modified Eagle medium
FDR: False discovery rate
GO: Gene Ontology database
HFF: Human foreskin fibroblast
IFA: Immunofluorescence assay
Kac: Lysine acetylation
KEGG: Kyoto Encyclopedia of Genes and Genomes
Kla: Lysine lactylation
KOG: Eukaryotic Orthologous Groups
LC–MS/MS: Liquid chromatography–tandem mass spectrometry
PASEF: Parallel accumulation serial fragmentation
PPI: Protein–protein interaction
PTM: Posttranslational modification
WB: Western blotting
