# Supplementary figures and images for: Systematic identification of the lysine lactylation in the protozoan parasite Toxoplasma gondii
Source: Parasit Vectors. 2022 May 24;15:180. doi: 10.1186/s13071-022-05315-6 (PMC9131557; doi:10.1186/s13071-022-05315-6)

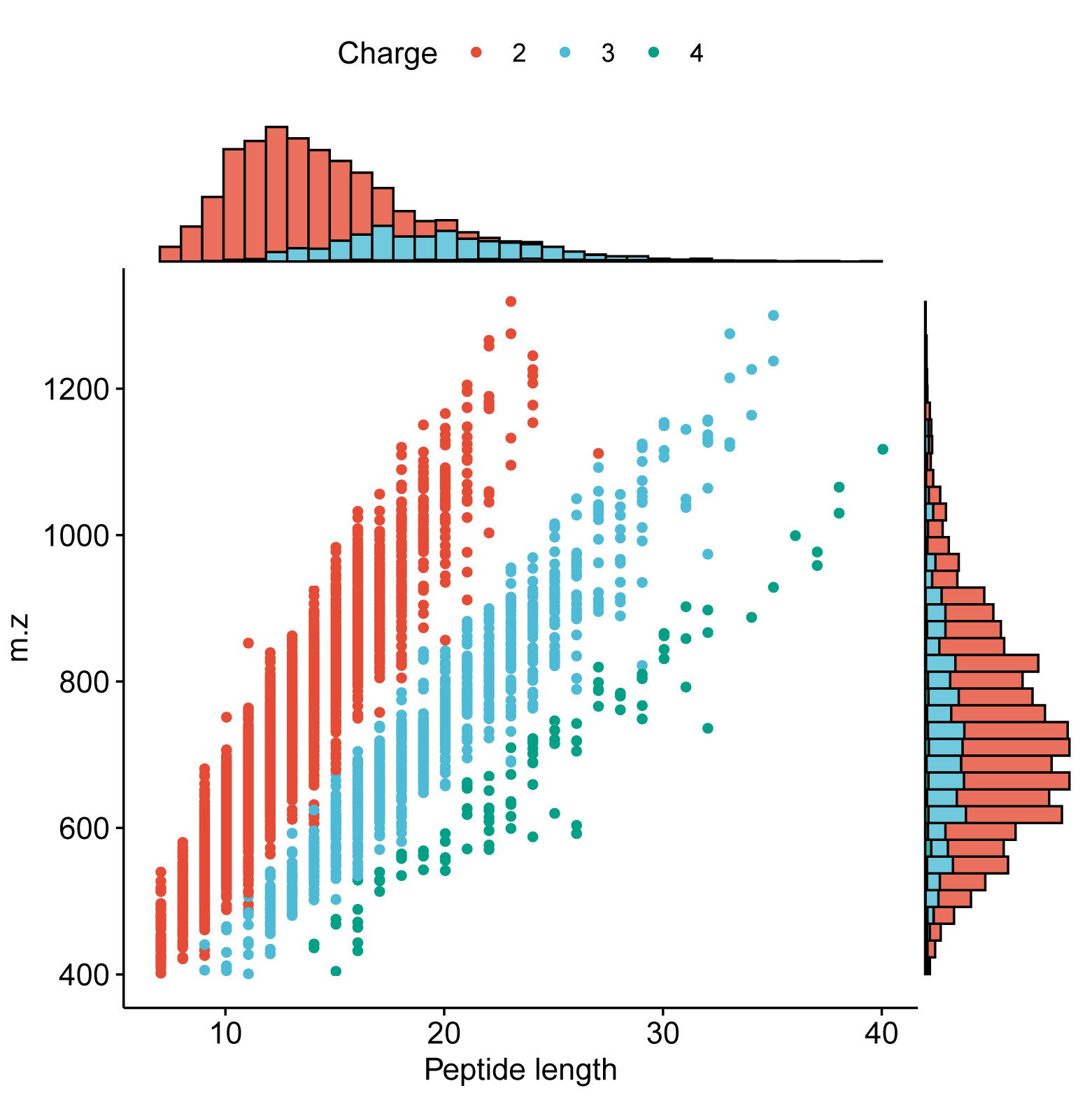

Supplement: Supplementary file 1 — Additional file 1: Fig. S1. Quality control of lactylated proteins in T. gondii. Scatter plot and bar plot show the peptide mass tolerance distribution and the length distribution of peptides identified by mass spectrometry, respectively. [file 13071_2022_5315_MOESM1_ESM.tif]
